# Supplementary figures and images for: Thermal comfort perception among park users in Prague, Central Europe on hot summer days—A comparison of thermal indices
Source: PLoS One. 2025 Jan 16;20(1):e0299377. doi: 10.1371/journal.pone.0299377 (PMC11737675; doi:10.1371/journal.pone.0299377)

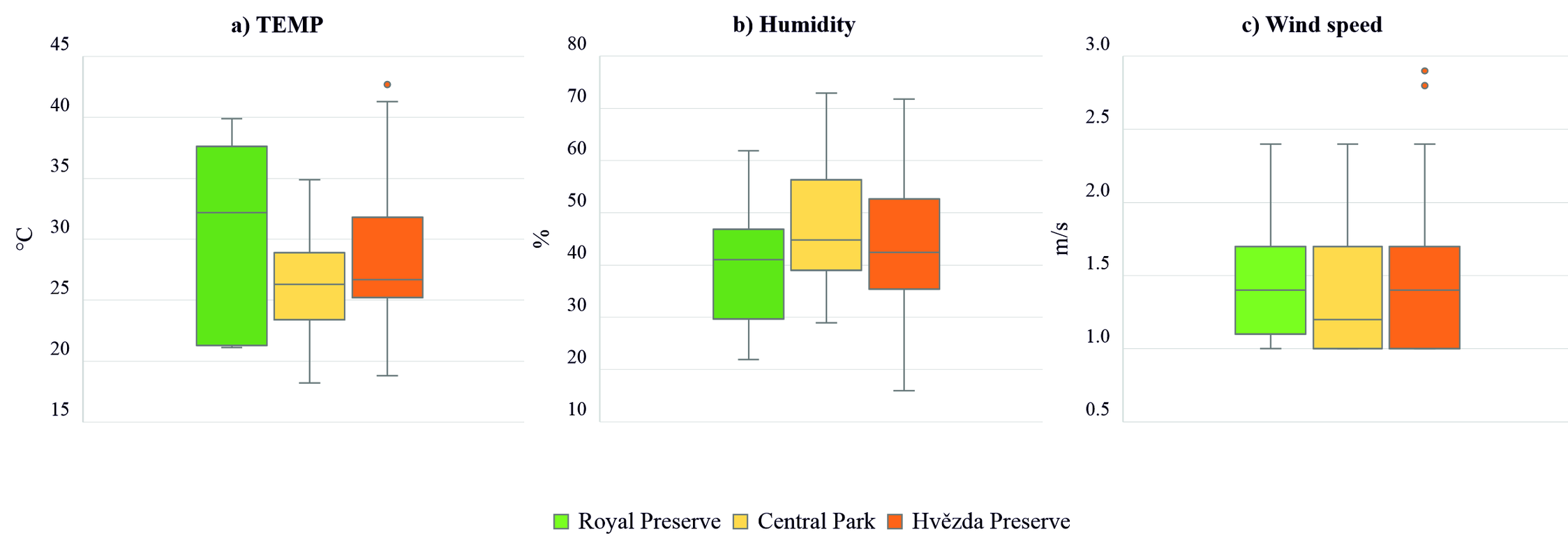

Supplement: S1 Fig — (TIF) [file pone.0299377.s001.tif]
