# Supplementary material for: Thermal comfort perception among park users in Prague, Central Europe on hot summer days—A comparison of thermal indices
Source: PLoS One. 2025 Jan 16;20(1):e0299377. doi: 10.1371/journal.pone.0299377 (PMC11737675; doi:10.1371/journal.pone.0299377)
Supplement: Attachment — Submitted filename: Comments.docx [file pone.0299377.s003.docx]

**General comments:** I appreciate the author's efforts on the critical issue of selecting appropriate outdoor heat indicators for architects and planners in Central Europe. This article is a comprehensive and typical study of thermal comfort in Central Europe, as it comprehensively evaluates the applicability of 4 commonly used thermal indicators and considers the influence of multiple factors (gender/age/visit time/activity level, etc.) on the applicability of the indicators. However, there are still some errors in this article that require some modifications.

1. There are some errors in the number of respondents in Table1, e.g., in the Royal Preserve column, the total number of respondents is 187 when differentiated by week and weekend or men and women. But when differentiated by morning, afternoon and evening, the total number is 176. The same error occurs in the other two parks' respondent counts, so be sure to recheck the numbers and correct them.

2. In Fig.1, satellite images or aerial photos of the three parks can be added to help readers understand more detailed information about the sites (area, green space coverage, and surroundings, etc.).

3. In line 288, consider including a correlation analysis between wind speed and visitors' heat perception polls to determine if wind speed has an effect on visitors' heat perception? If the correlation is not strong, it suggests that the failure to account for changes in wind speed is the reason why the HI index is more competent for evaluating thermal comfort in Central European parks. A method for predicting the effect of a single meteorological factor on thermal perception can be found in this article: Factors influencing resident and tourist outdoor thermal comfort: A comparative study in China's cold region.

References:

Yu Tian, Bo Hong, Zhenqi Zhang, Shuang Wu, Tingting Yuan, Factors influencing resident and tourist outdoor thermal comfort: A comparative study in China's cold region, Science of The Total Environment, Volume 808, 2022, 152079, ISSN 0048-9697, https://doi.org/10.1016/j.scitotenv.2021.152079.

4. In line 322, people's tolerance for heat is higher at night than during the day, mainly because nighttime does not have the strong solar radiation of daytime, which acts directly on the temperature receptors on the skin to produce a stronger sense of heat. Of course, there can also be some influence of psychological factors such as thermal history that lead tourists to perceive hotter environments as more comfortable at night. You can refer to this article: Perception, physiological and psychological impacts, adaptive awareness and knowledge, and climate justice under urban heat: A study in extremely hot-humid Chongqing, China.

References:

Bao-Jie He, Dongxue Zhao, Xin Dong, Ke Xiong, Chi Feng, Qianlong Qi, Amos Darko, Ayyoob Sharifi, Minal Pathak, Perception, physiological and psychological impacts, adaptive awareness and knowledge, and climate justice under urban heat: A study in extremely hot-humid Chongqing, China, Sustainable Cities and Society, Volume 79, 2022, 103685, ISSN 2210-6707, https://doi.org/10.1016/j.scs.2022.103685.

5. Line 349, a) and b) should be used to illustrate the pictures in Fig. 6, not left or right.

6. Line 349, according to the results of Fig.6, it cannot be directly concluded that the middle-aged group can tolerate the heat better than the young people, but it can only be directly stated that the middle-aged group can tolerate the heat better than the old people. If there is any data analysis that can support the conclusion that the middle-aged group is the group that can tolerate heat the best, please add it.
